# Radiation emergency preparedness in the middle east: Public health consequences, water security vulnerabilities, and evidence-based countermeasures

**DOI:** 10.17179/excli2026-9450

**Published:** 2026-06-25

**Authors:** Helmi Ben Saad, Ismail Dergaa, Halil Ibrahim Ceylan, Andrea de Giorgio, Nicola Luigi Bragazzi

**Affiliations:** 1 Research Laboratory LR12SP09 'Heart Failure', Faculty of Medicine 'Ibn el Jazzar' of Sousse, Farhat Hached University Hospital, Sousse 4002, Tunisia; 2 Department of Physiology and Functional Explorations, Farhat Hached University Hospital, Sousse 4002, Tunisia; 3 High Institute of Sport and Physical Education of Ksar Said, University of Manouba, Manouba 2010, Tunisia; 4 Physical Activity Research Unit, Sport and Health (UR18JS01), National Observatory of Sports, Tunis 1003, Tunisia; 5 High Institute of Sport and Physical Education of Kef, University of Jendouba, Kef 7100, Tunisia; 6 Physical Education of Sports Teaching Department, Faculty of Sports Sciences, Atatürk University, Erzurum 25240, Türkiye; 7 Artificial Engineering, Naples 80121, Italy; 8 Laboratory for Industrial and Applied Mathematics (LIAM), Department of Mathematics and Statistics, York University, Toronto, ON M3J 1P3, Canada; 9 Department of Clinical Pharmacy, Saarland University, 66123 Saarbrücken, Germany

**Keywords:** disaster planning, emergency management, emergency medical services, environmental exposure, nuclear warfare, public health, risk assessment

## Abstract

The ongoing military conflict involving the United States, Israel, and Iran has transformed long-theorized nuclear emergency scenarios into credible near-term threats. Iran's Bushehr Nuclear Power Plant, located on the Persian Gulf coastline just 17 km from the city of Bushehr, lies closer to several Gulf Cooperation Council capitals than to Tehran. At the same time, Qatar, the United Arab Emirates, Kuwait, Bahrain, and Saudi Arabia depend on seawater desalination for 60-90 % of their freshwater supply, with strategic reserves typically lasting between 36 hours and seven days. This convergence of nuclear proximity and water dependency creates a compound public health risk insufficiently addressed in the current literature. This review aimed to characterize plausible nuclear and radiation threat scenarios in the Middle East, map the spectrum of public health consequences from immediate blast and thermal injuries to long-term genetic damage, examine the risk of radiation contamination of the Persian Gulf leading to regional water supply collapse, and propose an evidence-based preparedness framework. A narrative review of peer-reviewed studies, international agency reports, and governmental guidance was conducted using MEDLINE/PubMed, Web of Science, Scopus, and grey literature from major global health and nuclear safety organizations. A single nuclear detonation in a major Gulf city could result in hundreds of thousands of immediate casualties while rendering seawater unusable for desalination over a prolonged period. Freshwater reserves would be rapidly depleted, leading to the simultaneous collapse of healthcare services, food systems, and sanitation infrastructure. Children and fetuses represent the most vulnerable groups, with increased risks of thyroid cancer, neurodevelopmental damage, and heritable mutations following radiation exposure. Key preparedness strategies include potassium iodide prophylaxis, shelter-in-place protocols, expansion of water reserves to at least 90 days, and strengthened regional civil defense coordination. Preparing for low-probability, high-impact events is far less costly than the consequences of inaction.

See also the graphical abstract(Fig. 1).

## Abbreviations

DTPA: Diethylenetriamine Pentaacetic Acid

FDA: Food and Drug Administration

GCC: Gulf Cooperation Council

Gy: Gray (unit of absorbed radiation dose)

IAEA: International Atomic Energy Agency

KI: Potassium Iodide

PTSD: Post-Traumatic Stress Disorder

RO: Reverse Osmosis

UAE: United Arab Emirates

WHO: World Health Organization

## Introduction

The atomic bombings of Hiroshima and Nagasaki in August 1945 killed between 110,000 and 210,000 people within the first four months and irradiated hundreds of thousands more, establishing with irrevocable clarity that nuclear weapons occupy a category of harm unmatched by any conventional armament (Glasstone and Dolan, 1977[20]; United Nations Scientific Committee on the Effects of Atomic Radiation, 2008[52]). Eight decades later, nine states collectively possess approximately 12,500 nuclear warheads, and the geopolitical architecture that once constrained their use has eroded at a pace that has alarmed every major international body (Kristensen and Korda, 2023[37]; World Health Organization, 2006[64]). The International Committee of the Red Cross has formally declared that no credible humanitarian response to a nuclear detonation is possible, a position that the World Health Organization (WHO) and the International Physicians for the Prevention of Nuclear War have each endorsed through separate, independent analyses (International Committee of the Red Cross, 2023[30]; International Physicians for the Prevention of Nuclear War, 2013[31]). Rather than being alarmist, this consensus is the product of 70 years of careful scholarship applied to the medical records of atomic bomb survivors, Chernobyl liquidators, and nuclear test veterans, and it converges on a single operational conclusion: prevention and preparedness are the only viable public health tools that exist (Boulton and Dunn, 2020[5]; Abbasi et al., 2025[1]).

The Middle East presents a convergence of radiation risk factors that no other inhabited region of the earth matches (Dallas and Burkle, 2011[13]). Iran operates a 1,000-megawatt electric nuclear power reactor directly on the shore of the Persian Gulf, 17 km southeast of the city of Bushehr, between the fishing villages of Halileh and Bandargeh (World Nuclear Association, 2025[66]). The reactor lies closer to Manama, Doha, Kuwait City, Abu Dhabi, and Muscat than it does to Tehran, meaning that a radiation release from Bushehr would reach Gulf Cooperation Council (GCC) capitals before it reached the Iranian capital (World Nuclear Association, 2025[66]; Global Energy Monitor, 2025[21]). Israel is independently estimated to maintain 90 nuclear warheads deliverable by land-based ballistic missiles, aircraft, and submarine-launched platforms, a capability that has been described in technical literature for decades (Kristensen and Korda, 2021[36]). The military escalation that began on 28 February 2026 brought these previously theoretical risks into proximity with operational reality: Russia's state nuclear corporation Rosatom publicly warned on 3 March 2026 that the Bushehr facility faces a direct threat as explosions have been confirmed within kilometers of the plant. The scientific community cannot afford to treat these developments as background noise.

Despite the documented scale of nuclear public health risks, critical gaps in the preparedness literature persist, particularly in the Middle East (Farhat et al., 2024[18]). Published scenario analyses modelling nuclear exchange in the region, including the rigorous 2013 study in Conflict and Health, focused primarily on blast radius casualties and fallout dose estimates but did not address water infrastructure collapse as an independent catastrophic pathway (Cham and Kim, 2013[9]). The WHO's foundational 1987 report on nuclear war and health predates the commissioning of the Bushehr plant and the current desalination dependency of GCC states (World Health Organization, 1987[63]) entirely. No peer-reviewed study has yet modelled the compound scenario in which radiation contamination of the Persian Gulf, a semi-enclosed body of water with a mean depth of only 36 m and restricted oceanic exchange through the Strait of Hormuz, forces the simultaneous shutdown of desalination plants across six nations whose populations hold freshwater reserves of fewer than seven days (Reynolds, 1993[45]; Johns et al., 2003[32]). Rather than being a minor lacuna, this gap is a structural blind spot in global emergency planning that could expose a single unanticipated event to catastrophic consequences and irreversibility. National preparedness frameworks across the GCC have historically prioritized oil infrastructure protection and conventional terrorism response, while radiation mass-casualty planning, including potassium iodide stockpiling, radiation triage training, and emergency water contingencies, remains conspicuously absent from publicly available civil defense documentation in most member states (Kemp and Grossman, 2024[34]; United Nations Office for the Coordination of Humanitarian Affairs, 2022[50]).

**Figure 1:**
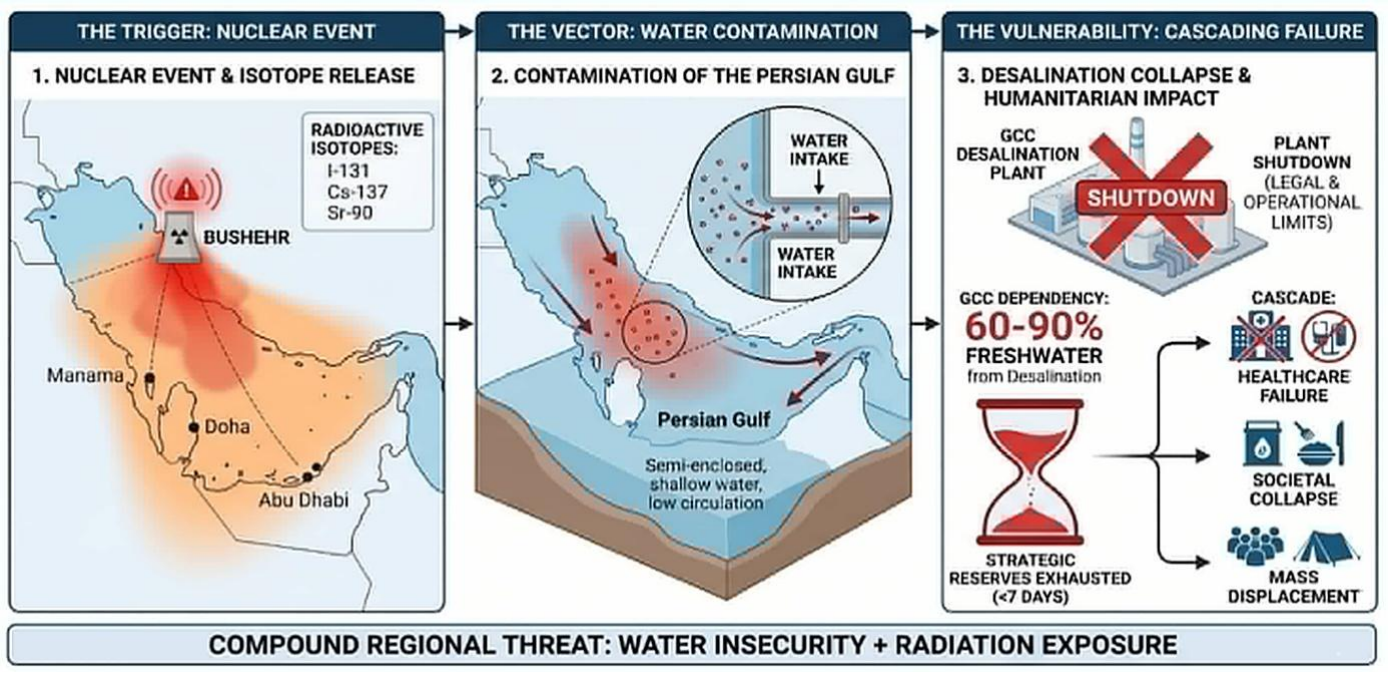
Graphical abstract: Compound public health risk in the Persian Gulf: Nuclear event trigger, water contamination vector, and cascading failure vulnerability. Cs-137, Caesium-137; GCC, Gulf Cooperation Council; I-131, Iodine-131; Sr-90, Strontium-90

Against this backdrop, the present review was developed to serve one clear scientific purpose: evidence-based preparation reduces harm regardless of whether the threat fully materializes, and the costs of preparation are trivially small relative to the costs of unpreparedness. This paper examined ***(i)*** the plausible typology of nuclear and radiation threat scenarios in the current Middle East context, ***(ii)*** the full spectrum of public health consequences from immediate mass-casualty events to multigenerational biological injury, ***(iii)*** the specific and underappreciated threat pathway from radiation contamination of the Persian Gulf to water supply collapse in GCC states, and ***(iv)*** an evidence-based preparedness framework spanning individual pharmacological countermeasures, clinical response protocols, and national policy recommendations. The analysis does not assume that these events will occur and holds that scientific preparedness is the highest expression of public health responsibility. The conceptual framework underlying this review is summarized in Figure 1(Fig. 1).

## Threat Scenarios: A Typology of Nuclear and Radiation Risks

### Nuclear detonation by state actor

The most consequential scenario involves the deliberate detonation of a nuclear weapon by a state actor targeting a population center. Yields relevant to current Middle East arsenals range from 15 kilotons, comparable to the Hiroshima device, to 150 kilotons for modern tactical nuclear weapons (Glasstone and Dolan, 1977[20]; Buddemeier et al., 2011[8]). A 15-kiloton airburst over a city of two million people would create a fireball zone with near-total destruction within a 1 km radius, a severe damage zone extending to 3 km, and a moderate damage zone to 6 km, with thermal flash burns affecting exposed populations to distances of 10 km or beyond (Glasstone and Dolan, 1977[20]; US Department of Homeland Security, 2010[54]). Immediate casualties would number in the hundreds of thousands. The fallout plume, shaped by prevailing winds, would carry radioactive contamination downwind for distances of 50 to 300 km, depending on weapon design and meteorological conditions (Glasstone and Dolan, 1977[20]; US Department of Homeland Security, 2010[54]). In the compact geography of the Persian Gulf, this plume would routinely cross-national borders within hours.

### Radiation dispersal device

A radiation dispersal device, commonly called a dirty bomb, combines conventional explosives with radioactive material to contaminate a defined area without producing a nuclear yield. This scenario carries a higher probability than deliberate nuclear weapon use, requires far less technical sophistication, and exploits the documented vulnerability of radioactive source materials in industrial and medical facilities across conflict zones (Mettler and Voelz, 2002[40]; IAEA, 2003[26]). Caesium-137, cobalt-60, and strontium-90 represent the most hazardous agents due to their high activity, long half-lives, and established dispersal characteristics (International Atomic Energy Agency, 2003[26]; Desai et al., 2021[15]). The public health consequences are more from long-term environmental contamination, psychological mass panic, and the economic destruction of affected commercial districts than from acute radiation exposure, which would be modest in most realistic dispersal scenarios. In a port or coastal city, maritime dispersal directly into the Persian Gulf remains a credible pathway for contamination of desalination water intakes.

### Nuclear reactor damage under conflict conditions

The Bushehr scenario represents perhaps the most plausible radiation threat pathway in the current conflict. A reactor operating at full power that sustains damage to its cooling system, regardless of whether the damage is caused by a direct military strike, blast overpressure from a nearby detonation, or cyber interference with safety systems, will progress through core heating to fuel cladding failure and, in the worst case, core meltdown with atmospheric and aquatic release of radioactive fission products (International Atomic Energy Agency, 2015[27]; Lyman, 2016[39]). The 2011 Fukushima Daiichi accident demonstrated that even a well-designed modern reactor could release caesium-137, iodine-131, strontium-90, and tritium into both the atmosphere and adjacent water bodies when cooling is lost under crisis conditions (International Atomic Energy Agency, 2015[27]). Bushehr is not cooled by the open ocean: it draws cooling water from the Persian Gulf and returns heated effluent to the same enclosed body of water. A release into that system would be amplified by the Gulf's restricted circulation, potentially persisting for years rather than months (Reynolds, 1993[45]; Johns et al., 2003[32]). The International Atomic Energy Agency (IAEA)'s nuclear watchdog confirmed in early March 2026 that no damage to nuclear facilities had been detected at that point (International Atomic Energy Agency, 2026[29]). The emphasis belongs to the phrase “at that point”.

## Immediate and Short-Term Public Health Consequences

### Blast and thermal injuries

The immediate physical effects of a nuclear detonation can be divided into three simultaneous insults: the *blast wave*, *thermal radiation*, and *prompt ionizing radiation*. The blast wave travels at supersonic speed, producing a positive-pressure phase that destroys structures and a negative-pressure phase that pulls debris outward at lethal velocity (Glasstone and Dolan, 1977[20]; Hall and Giaccia, 2019[23]). Lung injuries, ruptured eardrums, and the penetrating trauma of glass and building fragments account for a substantial proportion of near-field casualties. Thermal radiation travels at the speed of light, meaning exposed individuals receive burns before they can react. Estimates from nuclear test data indicate that approximately half of unprotected persons outdoors at one mile from a 10-kiloton airburst would sustain potentially fatal third-degree burns (US Department of Homeland Security, 2010[54]; Hall and Giaccia, 2019[23]). The firestorm phenomenon, in which individual fires coalesce into a self-sustaining conflagration, imposes an additional oxygen-depletion and carbon monoxide hazard that renders even subterranean shelters lethal if inadequately sealed.

### Acute radiation syndrome

Prompt ionizing radiation at doses exceeding 1 Gray (Gy) triggers acute radiation syndrome, a time-sequenced clinical cascade that proceeds through four recognized stages: prodrome, latency, manifest illness, and recovery or death (Waselenko et al., 2004[60]; Dainiak, 2002[12]). At whole-body doses between 2 and 6 Gy, the hematopoietic syndrome dominates, characterized by severe neutropenia and thrombocytopenia that manifest 10 to 14 days after exposure and produce death from infection and hemorrhage in the absence of bone marrow supportive care (Waselenko et al., 2004[60]; Dainiak, 2002[12]). Above 6 Gy, gastrointestinal syndrome emerges with intestinal crypt cell death, fluid and electrolyte loss, and translocation of enteric bacteria. Above 10 Gy, the neurovascular syndrome produces rapid death from cerebral oedema regardless of treatment (Waselenko et al., 2004[60]). Critically, anyone outdoors within approximately 1.5 km of a 10-kiloton detonation and in direct line of sight would receive a dose in the 3 to 6 Gy range, sufficient to produce severe hematopoietic syndrome that carries a case fatality rate of 50 % without medical intervention (US Department of Homeland Security, 2010[54]; Dainiak, 2002[12]).

### Collapse of healthcare infrastructure

Nuclear weapon effects on healthcare infrastructure are systematic, not incidental. Hospitals, clinics, and emergency services are concentrated in precisely the urban areas that represent high-value nuclear targets. Published estimates for a 10-kiloton detonation in a large US city modelled 630,000 patients requiring 352,000 hospital beds, 42,000 burn beds, and 134,000 intensive care beds against a background of 1,333 burn beds and 61,000 intensive care beds available (National Academies of Sciences, 2009[42]). Middle Eastern cities, with generally lower hospital bed-to-population ratios, would face even more extreme resource gaps. Blood product shortfalls would be catastrophic: 12,000 units of platelets are potentially available against 15 million required (National Academies of Sciences, 2009[42]). Physicians and first responders cannot operate effectively in radioactively contaminated areas without dosimetry, protective equipment, and decontamination facilities that no country in the region currently holds at the necessary scale.

### Mass psychological injury

Psychological casualties from a nuclear or radiation event routinely outnumber physical casualties by an order of magnitude, as demonstrated by the aftermath of Chernobyl, the Three Mile Island incident, and the 2011 Fukushima accident (Bromet and Havenaar, 2007[7]; Hasegawa et al., 2015[24]). Post-traumatic stress disorder (PTSD), depression, anxiety disorders, and medically unexplained somatic complaints afflict exposed and non-exposed populations alike, driven by radiation anxiety that persists for decades after the acute event. Following Chernobyl, population-level surveys documented elevated rates of depression and PTSD across Belarus, Ukraine, and Russia for at least 25 years post-accident (Bromet and Havenaar, 2007[7]). In a region already experiencing active armed conflict, the psychological burden of a nuclear or radiation incident would layer onto pre-existing trauma exposure, with consequences for mental health infrastructure that are almost impossible to fully model in advance.

## The Persian Gulf Water Crisis: Desalination, Contamination, and Cascading Failure

### The scale of desalination dependency in GCC states

The water security vulnerability of GCC nations is quantitatively unlike anything else in the world. There are no permanent rivers or natural lakes in Saudi Arabia, Bahrain, Kuwait, the United Arab Emirates (UAE), Qatar, Yemen, or Oman (Al-Zubari, 1998[3]; Hakimdavar, 2024[22]). Fresh groundwater aquifers, formed by Pleistocene-era rainfall, are non-renewable at any human-relevant timescale and have been drawn down to near depletion by agricultural extraction (Al-Zubari, 1998[3]). Desalination of seawater provides 90 percent of Kuwait's drinking water, 86 percent of Oman's, between 75 and 90 percent of Qatar's, and 40 to 70 percent of Saudi Arabia's, with the UAE and Bahrain each in comparable ranges (Hakimdavar, 2024[22]; Moody's Investors Service, 2025[41]). Qatar's strategic freshwater reserve amounts to seven days of supply under normal consumption conditions (Alyaseri et al., 2021[2]). Earlier reports from the same body of literature placed the reserve as low as 36 hours if desalination plants were to stop operating (Russian International Affairs Council, 2021[46]). These figures define the window within which a humanitarian collapse would unfold if Persian Gulf seawater were rendered unprocessable.

### Persian gulf hydrodynamics and contamination persistence

The Persian Gulf is a semi-enclosed shallow sea approximately 990 km long with a mean depth of 36 m and restricted exchange with the open Indian Ocean through the Strait of Hormuz (Reynolds, 1993[45]; Sheppard et al., 1992[48]). Its hydrodynamic characteristics mean that water masses and dissolved contaminants circulate within the Gulf for months before any significant dilution by open-ocean exchange occurs (Sheppard et al., 1992[48]). Radioactive caesium-137, with a physical half-life of 30 years, strontium-90 with a 29-year half-life, and iodine-131 with an 8-day half-life, would each behave differently in this environment: iodine-131 would decay relatively quickly, while caesium-137 and strontium-90 would persist in Gulf waters for years, contaminating the seawater intakes of hundreds of desalination plants simultaneously (Desai et al., 2021[15]; Hirose et al., 2004[25]). Standard reverse osmosis (RO) membranes, which underpin most GCC desalination technology, are effective at removing some but not all dissolved radionuclides. Caesium-137 passes through RO membranes at removal efficiencies that fall well short of the safety thresholds for drinking water (Kim et al., 2018[35]).

### The cascade from intake shutdown to humanitarian collapse

When desalination plant operators detect radioactive contamination above regulatory thresholds in their seawater intakes, they are legally and operationally required to shut down production. The 2008 red tide bloom in the Gulf demonstrated that a biological contamination event could force the simultaneous shutdown of desalination plants across multiple GCC states for weeks (Alyaseri et al., 2021[2]). A radiation contamination event would trigger the same protocol, but the cause would not be cleared in weeks: it would persist for months to years, depending on the radionuclides involved.

**Figure 2:**
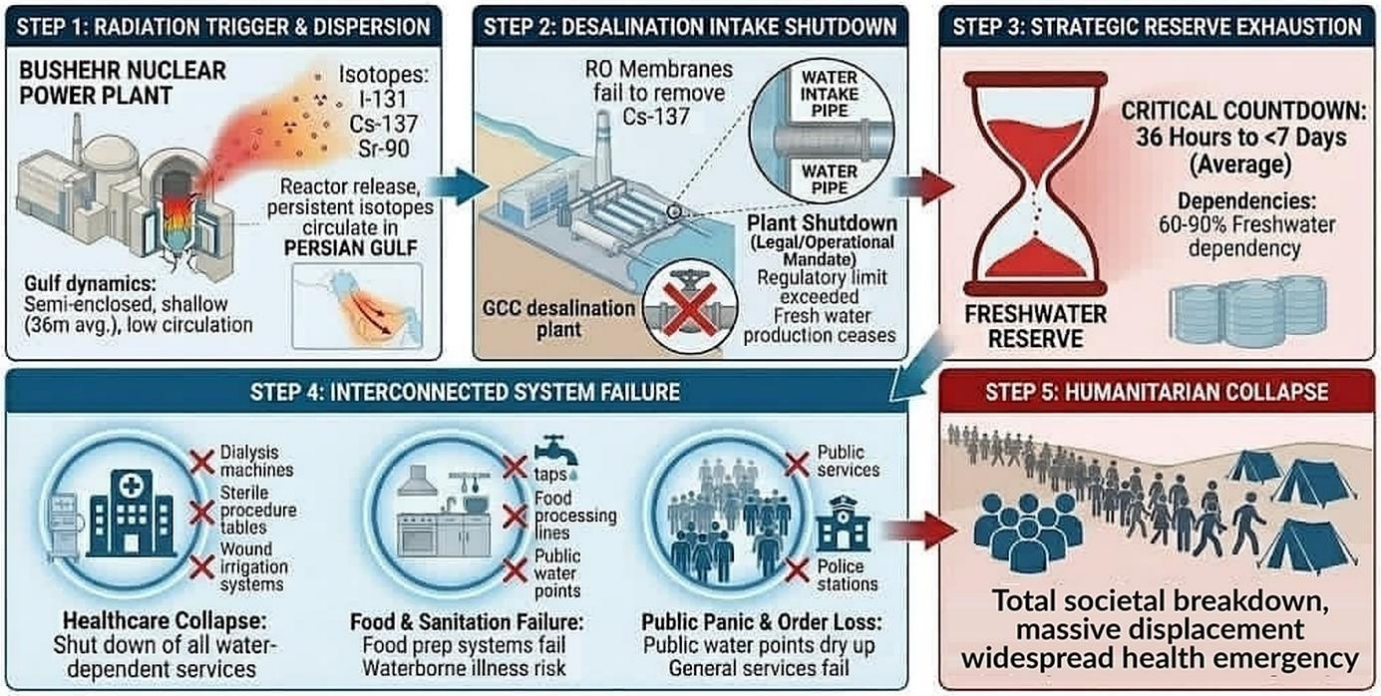
The five-step cascading failure pathway from Bushehr nuclear release to regional humanitarian collapse via desalination shutdown in Gulf Cooperation Council (GCC) states. Avg, Average; Cs-137, Caesium-137; I-131, Iodine-131; RO, Reverse Osmosis; Sr-90, Strontium-90

Within Qatar's documented 36-hour to seven-day reserve window, the following sequential failures would unfold: drinking water supplies exhausted, then hospital water for sterile procedures, wound irrigation, and dialysis; then food preparation and sanitation systems failing; then mass population panic and flight. A city of three million people with no water supply and no credible restoration timeline represents a public health emergency of a scale for which no precedent or preparedness plan exists in the existing literature. The economic and social disintegration would be total and rapid (Figure 2(Fig. 2)).

## Pediatric and Reproductive Health: The Multigenerational Burden

### Thyroid cancer and radioiodine exposure in children

Children represent the most radio-biologically vulnerable subgroup in any nuclear or radiation event, and the Middle East's young demographic profile amplifies this risk substantially. Qatar, the UAE, and Saudi Arabia report youth dependency ratios between 19 and 29 percent, meaning that children under 15 constitute a large fraction of the population most likely to ingest radioiodine through contaminated water and food supplies (United Nations Population Fund, 2024[51]).

**Figure 3:**
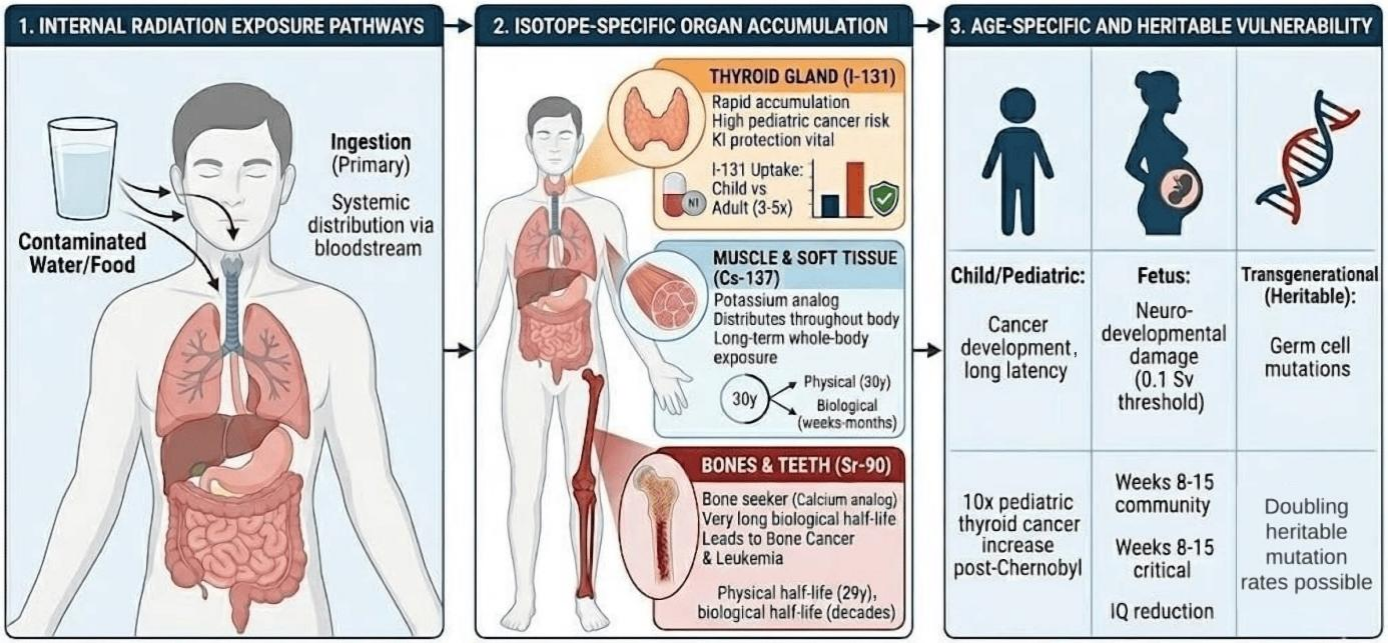
Internal radiation exposure pathways, isotope-specific organ accumulation, and age-dependent health consequences following ingestion of radioactive contamination. Cs-137, Caesium-137; I-131, Iodine-131; IQ, Intelligence Quotient; KI, Potassium Iodide; NT, Normal Thyroid; Sr-90, Strontium-90; Sv, Sievert; y, Year

The pediatric thyroid accumulates iodine at rates 3 to 5 times higher than the adult thyroid, and a developing thyroid gland exposed to iodine-131 at doses that produce no measurable effect in an adult will develop papillary thyroid cancer detectable within 3 to 10 years (Williams, 2002[62]; Kazakov et al., 1992[33]). The Chernobyl accident documented this relationship with devastating clarity: thyroid cancer incidence among children exposed to iodine-131 fallout increased more than tenfold in the most heavily affected regions of Ukraine and Belarus within five years of the accident (Williams, 2002[62]; Saenko et al., 2011[47]). A comparable release into the Persian Gulf would deliver radioiodine through contaminated water and food chains to children across six GCC nations, with effects emerging in the pediatric oncology burden for at least a decade after the event (Figure 3(Fig. 3)).

### Fetal brain development and the critical gestational window

The developing fetal brain is uniquely sensitive to ionizing radiation during a specific and identifiable gestational window (Verreet et al., 2016[59]). Whole-body doses as low as 0.1 Gy delivered during gestational weeks 8 to 15 produce measurable and dose-dependent reductions in intelligence quotient, with severe mental disability confirmed in children born to mothers within 2 km of the Hiroshima and Nagasaki detonation points (Otake and Schull, 1984[43]). This was not a marginal statistical signal. Within a decade of the atomic bombings, researchers had documented small head size, significant functional impairment, and substantially poor performance on intelligence quotient assessments in children whose mothers had been gestating at the time of exposure (United Nations Scientific Committee on the Effects of Atomic Radiation, 2014[53]). Radiation doses deliverable through drinking water contaminated by long-lived radionuclides, even at sub-lethal chronic exposure levels well below those required to produce acute radiation syndrome, fall within the range associated with measurable neurodevelopmental impairment in exposed fetuses. The women of childbearing age in GCC populations who rely on desalinated water as their only freshwater source thus face a reproductive health risk that extends across generations and cannot be mitigated after the fact.

### Multigenerational genetic consequences

Beyond the direct health consequences in exposed individuals, ionizing radiation damages DNA in ways that can be transmitted to subsequent generations. Studies of the children of Chernobyl liquidators, using highly sensitive mini-satellite mutation assays, documented a statistically significant doubling of heritable mutation rates in children born within two years of parental exposure to Chernobyl fallout (Dubrova et al., 1996[16]). Animal studies confirm that radiation-induced heritable mutations persist across at least two post-exposure generations, with varying expression patterns that depend on the specific *loci* affected and the developmental stage at exposure (Little, 2003[38]). For a region in which Gulf states collectively house a population of approximately 50 million people, many of whom would be exposed to low-level radioactive contamination through water and food for years following a radiation event, the multigenerational public health consequence is an enduring and largely unquantified burden that will persist in cancer registries, developmental disability statistics, and reproductive health data for 30 to 50 years.

## Evidence-Based Preparedness Strategies

### Potassium iodide prophylaxis: Mechanism, efficacy, and dosing

Potassium iodide (KI) represents the best-studied and most deployable pharmacological countermeasure available for radiation emergencies, and its mechanism is straightforward: by saturating thyroid tissue with stable iodine before radioiodine arrives, KI blocks the uptake of iodine-131 by the gland with an efficacy exceeding 90 percent when administered 24 hours before exposure and approximately 40 percent when administered within four to eight hours after exposure (World Health Organization, 2017[65]; Zanzonico and Becker, 2000[67]). The WHO recommends that KI be prepositioned and pre-distributed to all populations within at least 100 km of nuclear facilities, in sufficient quantities to cover a minimum of 10 days of prophylaxis (World Health Organization, 2017[65]). Given that Bushehr lies within 340 km of Doha, 310 km of Manama, and 420 km of Abu Dhabi, WHO guidance would place the entirety of Qatar, Bahrain, and the coastal UAE within the zone warranting KI pre-distribution. Current publicly available GCC civil defense documentation contains no evidence that this has been implemented. Weight-based dosing guidelines are clear and well-validated: adults and adolescents between 12 and 40 years should receive 130 mg, children aged 3 to 12 years should receive 65 mg, infants aged 1 month to 3 years should receive 32 mg, and neonates should receive 16 mg (World Health Organization, 2017[65]; US Food and Drug Administration, 2001[57]). Adults over 40 years require KI only when the expected thyroid dose is very high, given their low residual thyroid cancer risk relative to their elevated risk of iodine-induced thyroid dysfunction. KI protects exclusively against radioiodine. It provides no protection against external gamma radiation, caesium-137, strontium-90, plutonium, blast injury, or thermal burns, and must be understood as one component of a coordinated response, not a standalone solution.

### Additional medical countermeasures for internal contamination

For internal contamination with caesium-137, the Food and Drug Administration (FDA)-approved therapeutic agent is Prussian blue, formally known as ferric hexacyanoferrate (US Food and Drug Administration, 2008[58]). Administered orally at 3 g three times daily in adults, Prussian blue acts as an ion exchange resin in the gastrointestinal tract, interrupting the enterohepatic recirculation of cesium and reducing its biological half-life by 60 to 70 percent (US Food and Drug Administration, 2008[58]; Breitenstein and Palmer, 1989[6]). For internal contamination with transuranic elements, including plutonium and americium, chelation therapy with calcium or zinc DTPA (for diethylenetriaminepentaacetic acid) promotes urinary excretion and should be initiated within 24 hours of suspected exposure, with calcium DTPA preferred in the acute phase (US Food and Drug Administration, 2004[55]). Hematopoietic acute radiation syndrome in the 2 to 6 Gy range responds to granulocyte colony-stimulating factor therapy, with filgrastim and pegfilgrastim both FDA-approved for this indication under the animal rule, which permits approval based on animal efficacy data when human trials are ethically and practically impossible (US Food and Drug Administration, 2015[56]). In the most severe cases of bone marrow failure, allogeneic hematopoietic stem cell transplantation offers a therapeutic option for a small fraction of patients with complete marrow ablation and no significant combined injury from burns or blasts. The volume of such cases in a mass-casualty scenario would overwhelm transplant capacity by several orders of magnitude, reinforcing the primacy of prevention and early shelter-based dose reduction.

### Shelter-in-place: The most immediately accessible dose-reduction intervention

For the majority of the population who find themselves neither at the epicenter of a nuclear detonation nor in the immediate contamination zone of a reactor release, shelter-in-place offers dose reductions that can mean the difference between lethal and sub-lethal radiation exposure (Coleman et al., 2015[11]). The protection factor of a building, defined as the ratio of outdoor dose to indoor dose, varies substantially by construction type: the basement of a reinforced concrete building offers a protection factor of 200 to 1,000 times, interior rooms of a concrete multi-story building provide 10 to 40 times protection, while a wood-frame single-family home provides protection of only 1.5 to 4 times (Federal Emergency Management Agency, 2017[19]). Given that the GCC's building stock is dominated by concrete construction due to the climate, the region is structurally well-positioned to provide effective shelter if the population is educated and prepared. Residents should be directed to move to interior rooms, seal ventilation gaps with available materials, turn off all air conditioning and HVAC (for heating, ventilation, and air conditioning) systems, and remain in shelter for a minimum of 24 to 48 hours while fallout dose rates decline as short-lived radionuclides decay (Federal Emergency Management Agency, 2017[19]). Removing and double-bagging outer clothing upon returning from contaminated outdoor environments reduces surface contamination by approximately 80 percent. Showering with soap and water removes the remainder.

### Water security countermeasures

In the Gulf context, water security during a radiation emergency requires a layered strategy that acknowledges the absence of alternative water sources. Pre-stored bottled water held in sealed containers is safe for consumption indefinitely and should represent the first-line emergency water supply for all GCC households, with a minimum of 72-hour individual reserves recommended and 14-day reserves as a planning target. RO technology, which already underlies most GCC desalination capacity, can reduce dissolved radionuclide concentrations for certain species, and upgrading existing plant pre-treatment to include ion exchange resins specifically targeting caesium-137 and strontium-90 is technically feasible and would extend operational viability under contamination conditions (Kim et al., 2018[35]). Boiling water does not remove radioactive contamination and, in fact, concentrates it by reducing water volume, a critical public health communication point that must be disseminated clearly in advance. National strategic water reserves in Qatar, currently documented at seven days, should be expanded to a minimum of 90 days as a matter of national security, a target that Qatar's Water Security Mega Reservoirs Project has explicitly identified but not yet fully realized (Russian International Affairs Council, 2021[46]; Qatar News Agency, 2025[44]).

### Nutritional and antioxidant strategies

At the individual level, pre-event optimization of antioxidant status is a low-cost, low-risk preparedness measure with biological plausibility, though its evidence base is primarily derived from animal models and *in vitro* systems rather than human clinical trials in radiation scenarios (Singh and Seed, 2017[49]). Ionizing radiation generates reactive oxygen species that drive DNA strand breaks, lipid peroxidation of membrane lipids, and protein carbonylation (Weiss and Landauer, 2009[61]). Experimental evidence supports a radio-protective effect of several dietary antioxidants at pharmacological doses, including alpha-tocopherol (vitamin E), *N*-acetylcysteine, and selenium (Weiss and Landauer, 2009[61]; Citrin et al., 2010[10]). Adequate nutritional status at the time of radiation exposure, including sufficient dietary iodine to saturate the thyroid independently of pharmaceutical KI, represents a readily achievable baseline that public health messaging should address in advance. The consumption of foods naturally high in stable iodine, including fish and dairy products from non-contaminated sources, reduces the fractional uptake of radioiodine by the thyroid in proportion to the degree of pre-existing saturation (Zanzonico and Becker, 2000[67]).

### National and regional policy recommendations

**Figure 4:**
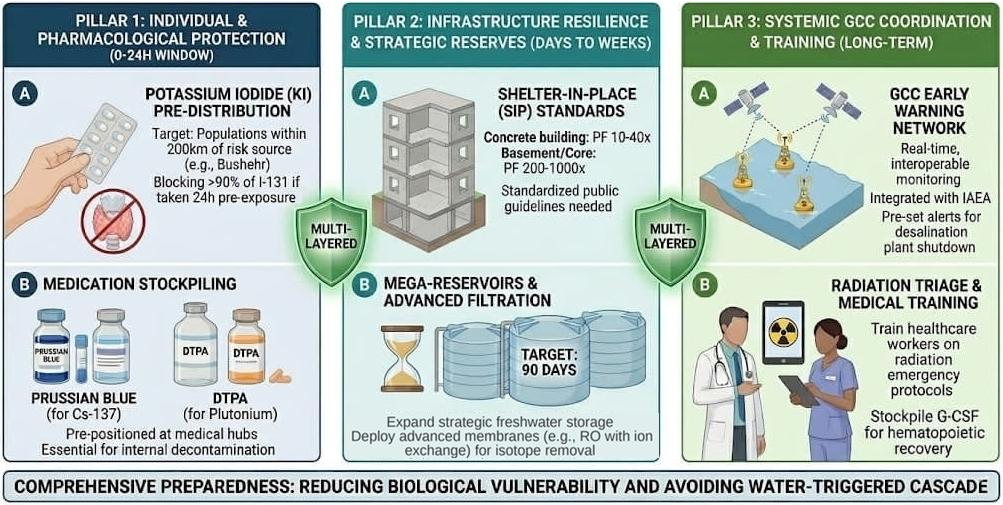
Three-pillar evidence-based preparedness framework for nuclear and radiation risks in the Gulf Cooperation Council (GCC) region: individual protection, infrastructure resilience, and regional coordination. DTPA, Diethylenetriamine Pentaacetic Acid; G-CSF, Granulocyte Colony-Stimulating Factor; I-131, Iodine-131; IAEA, International Atomic Energy Agency; PF, Protection Factor; RO, Reverse Osmosis

The evidence reviewed in the preceding sections supports a set of specific, actionable policy recommendations directed at Gulf state governments, regional bodies, and international organizations (Figure 4(Fig. 4)). These are offered not as speculative proposals but as evidence-grounded interventions with established precedents in countries that have developed mature nuclear emergency planning frameworks. Concerning the pre-distribution of potassium iodide, all GCC states should implement household pre-distribution of KI tablets to residents within a 200 km radius of the Bushehr Nuclear Power Plant, covering the entirety of Bahrain and Qatar, the coastal UAE, and significant portions of Kuwait and Saudi Arabia. The European Commission's operational guidelines for public KI use provide a directly applicable model for such a program (European Commission, 2004[17]). Public communication campaigns explaining when and how to take KI, and, critically, what it does and does not protect against, must accompany pre-distribution to prevent both under-use due to ignorance and harmful misuse from incorrect self-administration. Regarding the strategic water reserve expansion, Qatar's documented 36-hour to seven-day freshwater reserve represents a critical national security vulnerability, unacceptable considering the identified nuclear proximity risk. A national target of 90 days of strategic freshwater reserve, achievable through the expansion of the Mega Reservoirs Project and complementary investment in underground aquifer recharge, would provide sufficient time for international humanitarian water supply to be mobilized in the aftermath of a desalination shutdown event (Alyaseri et al., 2021[2]; Qatar News Agency, 2025[44]). All GCC states should adopt comparable reserve targets. Regional early warning and information sharing. A GCC-wide real-time radiation monitoring network, integrated with the IAEA's global “Incident and Trafficking Notification System” for nuclear event notifications, would provide the earliest possible warning of releases from Bushehr or any other regional source. The technical infrastructure for such a network is mature and has been deployed at the national level across Europe following Chernobyl. Regional deployment requires political coordination rather than technical innovation. Concerning the training of healthcare professionals in radiation triage, emergency physicians, nurses, and paramedics across GCC states require standardized training in the clinical recognition of acute radiation syndrome, the triage of combined injuries, and the appropriate use of medical countermeasures. The IAEA and WHO published detailed guidance for radiation triage training that has not been systematically integrated into GCC medical education curricula (International Atomic Energy Agency, 1998[28]). Incorporating radiation emergency medicine into the training requirements for emergency physicians and disaster medicine specialists represents a minimum threshold of preparedness. Finally, regarding the designation and preparation of sheltering facilities, high-density concrete buildings in all major GCC cities should be identified, mapped, and publicized as designated shelter locations, with basic supplies including bottled water, KI tablets, and sealed food stockpiles pre-positioned. The public should know where to go and what to do before a radiation event occurs. Preparedness communication through national media, schools, and community organizations should be treated as a routine public health activity rather than an exceptional crisis measure.

## Limitations

This review carries several limitations that require explicit acknowledgment. The rapidly evolving conflict situation means that the specific operational threat parameters described here may change substantially between manuscript preparation and publication, potentially rendering some scenario estimates either overstated or understated. The review does not present formal quantitative risk modelling with uncertainty bounds, which would require dedicated computational simulation tools such as the “Hazard Prediction and Assessment Capability” or similar consequence assessment frameworks, beyond the scope of a narrative review. Casualty and contamination estimates drawn from Hiroshima, Chernobyl, and Fukushima data are extrapolations to a Middle Eastern context with different population densities, building stock characteristics, climate conditions, and healthcare system capacities, all of which introduce uncertainty into any consequence estimate. The evidence base for individual nutritional and antioxidant countermeasures is predominantly derived from animal models, and the human clinical literature on non-KI pharmacological radioprotection remains limited. The water security analysis, while grounded in peer-reviewed hydrological data, does not account for the potential of emergency water imports or the activation of alternative supply chains, which might partially offset the timeline to humanitarian collapse in a well-resourced response scenario. These limitations do not invalidate the central findings. They indicate where quantitative modelling research should be directed as a matter of scientific priority.

## Conclusion

This review has examined the compound public health threats arising from the proximity of Iran's Bushehr Nuclear Power Plant to Gulf states whose populations hold fewer than seven days of freshwater reserves and whose children constitute between 19 and 29 percent of their total population, within a geopolitical environment in which nuclear risk has moved from theoretical to operationally plausible. The convergence of factors documented here, namely a functioning nuclear reactor on the shore of an enclosed sea, desalination infrastructure with near-zero redundancy, dense urban populations with no alternative water source, and an active regional armed conflict, creates a public health risk profile for which the existing global preparedness literature has no direct precedents and no adequate guidance. The four main conclusions of this analysis are as follows. First, a single nuclear detonation or major reactor release into the Persian Gulf would produce public health consequences measurable not in days or months but in decades, with multigenerational genetic, oncological, and neurodevelopmental burdens extending well beyond the populations directly exposed. Second, the shutdown of desalination water intakes across six GCC nations would represent a cascading catastrophe whose timeline is measured in hours to days, not weeks, and which would overwhelm emergency response capacity before international humanitarian assistance could be organized. Third, evidence-based countermeasures exist and are deployable now: potassium iodide pre-distribution, strategic expansion of the water reserve to a minimum of 90 days, shelter-in-place infrastructure designation, and healthcare professional training in radiation triage are all achievable within existing technical and financial capacities. Fourth, the purpose of this paper is not to predict a catastrophe. It is to ensure that if a catastrophe comes, it does not find the region's populations and public health systems unprepared. The scientific community has both the knowledge and the obligation to act in pursuit of that purpose. Preparedness cannot wait for certainty.

## Declaration

### Acknowledgments

The authors would like to express their sincere gratitude to the reviewer for their excellent feedback, which has substantially improved the quality of our review.

### Artificial Intelligence (AI) - assisted technology

In preparing this manuscript, the author used Claude (Anthropic, San Francisco, CA) on March 6, 2026, to assist in organizing research material, improving the clarity of selected passages, and refining the grammatical quality of sections of the text. The tool was used to enhance academic tone and check language quality. The authors did not use AI tools for data analysis, interpretation, or the generation of scientific conclusions. After using this tool, the authors thoroughly reviewed, edited, and validated all content and take full responsibility for the accuracy, integrity, and scientific validity of the work (Ben Saad et al., 2025[4]; Dergaa et al., 2024[14]). Furthermore, Nano Banana 2 (Google LLC, Mountain View, CA) was utilized to generate the initial visual baselines and conceptual layouts for the graphical abstract as well as the figures within the manuscript. Following this initial generation, these visual elements underwent extensive manual modification, post-processing, and scientific verification by the authors using Adobe Photoshop (Adobe Inc., San Jose, CA) and illustrations from the BioRender library (BioRender, Toronto, ON, Canada) to produce the final versions. The AI tool was strictly limited to preliminary artistic drafting, and the authors assume full responsibility for the accuracy, originality, and scientific integrity of the final visual representations.

### Conflict of interest

The authors declare no competing interests.

### Funding

This narrative review received no specific funding.

### Availability of data

This is a narrative review. All primary sources are cited in the reference list and are publicly accessible through their respective publishers or institutional repositories.

### Consent for publication

All authors approved the final version for publication.

### Ethics approval

Not applicable for this narrative review analyzing published literature and public statements.

### Author contribution

Conceptualization: H.B.S., I.D., H.İ.C., N.L.B.; Literature Search: I.D., H.İ.C., A.d.G., N.L.B.; Methodology: I.D., H.İ.C., A.d.G., N.L.B.; Validation: A.d.G., N.L.B.; Writing - Original Draft: H.B.S., I.D., H.İ.C., A.d.G., N.L.B.; Writing - Review & Editing: H.B.S., I.D., H.İ.C., A.d.G., N.L.B.
